# Notes of an European Tour

**Published:** 1846-02-01

**Authors:** F. H. Hamilton

**Affiliations:** Professor of Surgery in Geneva Medical College


### Notes of an European Tour.---No. 9.

Communicated for the Buffalo Medical Journal, by F. H. HAMILTON, M. D., Professor of Surgery in Geneva Medical College.

GENOA, ITALY.

I have spent three days at Genoa most delightfully, rambling up and down through its streets, palaces, churches, hospitals, &c. Strolling along the beautiful Strada Balbi I came to the "University," a large palace-like building, standing like the adjoining palaces, quite upon the street. I entered its magnificent vestibule alone, and was looking about for "the Swiss" who usually occupies a small lodge off from the vestibule, acting in the complex capacity of cobler, clothes-cleaner, porter and guide, when my eyes unexpectedly rested upon two fierce looking Numidian lions crouching upon each side of the head of the stairway; like Bunyan's Pilgrim, for a moment I hesitated which way to pass. In Italy, every where, marble has a wonderful soul. "The Swiss," coming to my relief, led me into the several halls appropriated to the different faculties, viz: Law, Theology, Philosophy, and Medicine; all of which, except the "Hall of Medicine," are large, well furnished rooms, and handsomely adorned with excellent pictures. In the room for public examinations, is a pulpit where the students are required to hold disputations before the examiners upon such subjects as are allotted to them. The "Hall of Medicine," where most of the Medical lectures are delivered, is a plain, dirty, ill-arranged room; the seats being mere benches and all on the same level, and could not, I should think, be made to accommodate more than 130—the porter however says there are 250 medical students.—A few dingy portraits and historical pictures hung against the walls—the portraits belong to distinguished Genoese medical men, but I failed to recognize among them a familiar name; where is Rasori, the great Genoese physician who died in 1837, and who by his writings mainly, subverted the doctrines of Brown, which had taken deep root throughout the north of Italy especially! His principal essay was issued in 1800, immediately after the famous seige of 60 days, sustained within the walls of Genoa by Massena, he having been himself an eye-witness of all its horrors.—When the gates were at length thrown open, 15,000 of the inhabitants had died of famine, or of disease engendered by it, and of the garrison, of 7,000 soldiers only 2,000 remained fit for service. Dr. Mosseno is Professor of Surgery, and Dr. Mazzini of Anatomy. I think it was the latter to whom "the Swiss" introduced me; a slovenly, snuff-taking old gentleman who was so deaf, or so badly understood my not very pure French, that I was forced after several efforts to give over all farther attempts to prosecute the acquaintance. The medical degress are conferred by a Senate of 12 Doctors, including the 7 Professors of the Faculty of Medicine. The Library and Museum attached to the University, are respectable.

The Hospital of Incurables is one of the several princely charities of this city. Dr. Costa conducted me through its wards and various apartments. The present number of inmates is 500, of whom 52 are mild cases of lunacy; when they become frantic and ungovernable, they are sent to the large lunatic asylum outside the walls. But I was surprised to find one, in a large room containing many invalids, chained to his bed, and whose constant howlings and shrieks, with his violent efforts to release himself must have greatly disturbed his sane but feeble companions. I could not see the propriety of even his temporary detention in such a place.

The Hospital of Pammatone, founded in 1430 by an eminent lawyer, Bartolomeo Bosco, is also a "palace" designed for sick foreigners as well as Genoese, and contains, on an average, from 600 to 1000 patients. It has also attached, a Foundling Hospital, where illegitimates are received. The number of these little aliens who can never know a tie of relationship, has at times, in this one establishment, exceeded 3000; the annual number of receptions being as high as 550, of which 200 are received unquestioned through the private revolving wheel, and this from a population of 114,000, of whom 20,000 are priests or nuns. There may be some truth in one clause of the old Tuscan proverb, that Genoa had "a sea, without fish, mountains without wood, men without faith, and women without virtue." The boys remain here until they are able to work, and the girls longer unless they can be properly provided for.—The Surgeons to the Hospital are Stephano Bignoni, Michele Gouillion, Enrico Ruini and Ferdinando Dellacella.

The Alms House, ("Albergo dei Poveri") pleasantly placed without the inner walls, was founded by a Nobleman, and has few parallels in Europe. It will accommodate 2000, and although generally well filled, it is every where neat and orderly. A premium is offered, to such young men of Genoa as are humanely disposed, in the way of a small dowry to the parties whenever a girl is married out of the Alms House.

The pauperism of this city, and indeed of Sardinia generally, is astonishing. In Genoa alone are about 2300 vagabonds who have no home or shelter, and there have been seasons of scarcity in whibh one-third of the population of the province were dependant upon charity. There are now in and about the city in addition to the hospitals and alms houses which I have enumerated, 15 "Conservatorie" or religious charities for females, and yet with all these, one-half the pauperism of Genoa seems to live in the streets. Yet the palaces are more numerous and more splendid than in any city of its size in the world, the churches are no where more gorgeous, the priests who attend at the Alms House alone receive $14,000 annually, the public works, including the docks, the light house and the prodigious walls which surround the city give evidence of a vast treasury, the soil of the neighboring hills is fertile, and well adapted upon its sunny slopes for the vine and olive, its two harbors are spacious and secure, and convenient for the commerce of Europe, Asia and Africa, but what is all this—the Government is a despotism and the King a superannuated tyrant.

The Lunatic Asylum, under the care of Drs Costa and Francisco Buffa. is also without the city and pleasantly situated, except that the grounds are rather low. It is composed of a round central, with radiating buildings, and is two stories high. It has now 288 inmates. The keeper told me that about 15 per cent were generally cured. The causes of lunacy were as follows: eight or ten, religious excitement, five, solitary vice, and most of the remainder were deranged in consequence of misery, love, or "*aqua vitae*" (alcohol.) Both men and women, I am told, drink spirituous liquors in Genoa, although the native wines are excellent, abundant and cheap: such as become maniacal from this cause, seldom recover. I was pleased to learn that there is neither a dungeon, chain or straight jacket in the Asylum, force never being resorted to except in the most extreme cases. As I was passing out of one of the doors an opportunity was furnished me to see the application and effects of their non-coercive system; a maniac attempted to push by us and escape, and was so far successful as to gain the main hall, but being arrested here by a strongly bolted door he turned upon his keeper with furious menaces, who so far from resisting or attempting to restrain him by violence, immediately sat down and quietly waited for ten or fifteen minutes, until of his own accord he walked back into the gallery bowing and laughing as if it had all been intended as a mere joke.

Yesterday I accepted the invitation of Mr. Lester, the American Consul, to visit with him one of the palaces belonging to the Marquis Durazzo, especially to look over the library, which he has offered to sell to our Government. The son of the Marquis himself had the kindness to accompany us to the library. It contains about 12,000 volumes, some of which are beautifully illuminated; one, a printed volume, was dated 1460. The whole, including a large amount of valuable manuscripts will be sold at $30,000, which is scarcely more than the cost of the binding, the whole library having been rebound in a substantial manner at Paris only a few years since.

The evening I spent at Mr. Lester's with a small assembly of his friends, among whom were two Marquises in military costume, with their Marchionesses, a celebrated opera singer, an improvisatrice, a Surgeon and Master from a British War Steamer lying in port, and Dr. Botta, a physician of considerable reputation in Genoa and from the same family with Botta the Historian. Dr. Botta is weary of despotism, and sighs for the freedom of our land; he says "if I were younger I should certainly emigrate to America, but I have now a family and am too old, I must therefore forever relinquish the idea." He remarked that the press was literally in chains, that he having publicly ventured to express some doubts about certain points of discipline in the Lunatic Asylum, he had been "warned," and he did not know, suspected as he was of liberal feelings, at what moment he might be sent to the galleys.

The Salizzi palace, once the residence of Byron, the palace of the Doria family, the "Strada Nuova" or the street of palaces, the "vicoli" or narrow passages which thread the city like fissures in marble quarries, the sedan chairs and hand chariots upon which ladies are carried about, the donkeys and mules with their immense panniers and tinkling bells, the long lines of "facchini di carovana" or priviledged and *hereditary* porters who carry burdens to and from the warehouses, the street lined with shops glittering with silver and gold fillagree work or the street of the goldsmiths, the beggars, the barefooted friars, all constitute features in the picture of Genoa, of which I have no time now to speak. But the Duomo or Church of St. Lorenzo, is too rare to pass wholly unnoticed. Built mostly in the Saracenic style both within and without, its interior has given me some idea of oriental church architecture, being covered every where with carvings, pictures and costly guildings; its columns resting upon basaltic bases, are composed variously of granite, porphyry, pietra dura, and rich marbles of various colors, black and white, according to oriental custom, being most prevalent. No part of the church however equals the gorgous chapel of St. John the Baptist, where within an iron sarcophagus are kept the sacred dust, with a part of a finger, of the venerated saint! This chapel is shut against the female sex, save upon one day in the year; the great and liberal mind of Pope Innocent eighth having conceived this mode of avenging the ashes of the Saint upon the daughter of Herodias. Within the treasury is kept the "sacro catino", or as some say, the vessel in which Joseph received the blood as it flowed from the side of our Saviour! And regularly at appointed days during the year these sacred relics are carried before the starving, houseless, beggared, ignorant populace, and regularly are the coffers of the church filled and the bellies of the priesthood fattened.

By an ancient inscription upon this church you may learn that Genoa was founded by Janus I, the grandson of Noah! and that Janus II, Prince of Troy, afterwards took possession of it in the name of his ancestor. There is also a catholic tradition that, being destroyed, it was rebuilt in the days of Abraham, and again 1246 years B. C., and that 35 years after the death of Christ, Paul, Peter and Barnabas preached in this city.

The Ladies of Genoa may be seen in great numbers every pleasant evening walking upon the "acqua sola" a beautiful promenade upon the ancient ramparts. They are rather larger than the average of American ladies, with full black eyes, but destitute of expression; their features are regular and symmetrical, their forms stately and generally inclining to "en bon point," their walk remarkably easy and graceful. But what chiefly attracted my notice was the almost universal pallor, or what I would call dropsical transparency of their complexions, which may perhaps be ascribed to the exclusion of the air and the direct rays of the sun from their narrow and irregular streets, conjoined with the idle and luxurious mode of living of the higher classes. They seldom cover their heads with a bonnet, but wear, when out of doors, a long mantilla of white muslin, which falling from the head and forehead is fastened lightly under the chin, and from thence drops gracefully over the neck and shoulders to the waist.

To-morrow I am off, having taken passage in an American brig bound to Palermo, and if the Tyrrhean seas do not prove treacherous, you shall hear from me again from the rocks of Sicily.

